# High-Resolution Infection Kinetics of Entomopathogenic Nematodes Entering *Drosophila melanogaster*

**DOI:** 10.3390/insects11010060

**Published:** 2020-01-18

**Authors:** Alexis Dziedziech, Sai Shivankar, Ulrich Theopold

**Affiliations:** Department of Molecular Biosciences, The Wenner-Gren Institute (MBW), Stockholm University, 11419 Stockholm, Sweden; alexis.dziedziech@su.se (A.D.); sai.krishnamoorthy@su.se (S.S.)

**Keywords:** *Drosophila melanogaster*, kinetics, infection, entomopathogenic nematodes, wounding, *Heterorhabditis bacteriophora*, sepsis, septicemia, high-resolution microscopy

## Abstract

Entomopathogenic nematodes (EPNs) have been a useful model for studying wound healing in insects due to their natural mechanism of entering an insect host either through the cuticle or an orifice. While many experiments have shed light on nematode and host behavior, as well as the host immune response, details regarding early nematode entry and proliferative events have been limited. Using high-resolution microscopy, we provide data on the early infection kinetics of *Heterorhabditis bacteriophora* and its symbiotic bacteria, *Photorhabdus luminescens*. EPNs appendage themselves to the host and enter through the host cuticle with a drill-like mechanism while leaving their outer sheath behind. EPNs immediately release their symbiotic bacteria in the host which leads to changes in host behavior and septicemia within 6 h while EPNs travel through the host in a predictable manner, congregating in the anterior end of the host. This paper sheds light on the entry and proliferative events of EPN infection, which will further aid in our understanding of wound healing and host immune activation at a high spatiotemporal resolution.

## 1. Introduction

Nematodes are a diverse clade of organisms that infect many species, including vertebrates and invertebrates. Entomopathogenic nematodes (EPNs) are a natural threat to insect larvae and are of interest in the agricultural industry regarding pest control. EPNs parasitize Lepidopteran species as well as *Drosophila* larvae, which in turn serve as a model for understanding the host immune response against nematode infections in general, like in the case of Elephantiasis or Onchocerciasis. They enter the host through either the mouth or anus or use their hook-like tooth to burrow into the cuticle, past the epithelial layer to reach the hemocoel. Here, they are able to complete their life cycle, reproduce, and cause septicemia in the host through regurgitation of their symbiotic bacteria [1]. There are two genera of EPNs that are frequently used in larval infection assays, *Steinernema* and *Heterorhabditis*. Different species of *Heterorhabditis* and *Steinernema* are known to either ambush their hosts in order to gain access to the hemocoel, or actively seek their host, or “cruise” when the host is more likely to be sedentary [2,3]. In addition, *Steinernema* have been reported to be more pathogenic to the *Drosophila* host [4]. Perhaps one reason these EPNs have different infection strategies and pathogenicity is that they are not closely related and underwent divergent evolution, including their parasitization strategy [5,6].

EPNs form a complex necessary for proliferation after infection, which entails a symbiotic relationship with gram-negative proteo-gamma bacteria, such as *Photorhabdus* spp. and *Xenorhabdus* spp. in *Heterorhabditis* spp. and *Steinernema* spp. nematodes, respectively [7]. After entry, the worms regurgitate their bacteria into the host. Once bacteria proliferate, this signals to the dormant infective juvenile to differentiate into the sexual-stage adult. The bacteria release toxic secretions, which aid in the EPN complex invading the host, suppressing the immune function, and breaking down the internal tissues [8]. While bacteria provide nourishment for the nematode, axenic nematodes can successfully infect and kill host larvae, though with lower mortality rates [1]. It has also been documented in *Galleria* that the hemocytes do not attack invading EPNs and that PPOs are suppressed by the EPN complex, which can be seen in the decreased levels of melanization [9,10].

In the presence of EPNs, *Drosophila* larvae change their behavior, indicating their ability to detect the presence of external threats and engage in potentially protective behavior [11,12]. Nevertheless, EPNs can infect *Drosophila* either through cuticle or gut penetration, although it is unknown if there is a mode of entry that is more accessible or preferred for some EPNs, such as *Heterorhabditis*. However, when infected larvae are entered by an EPN via the cuticle, several pathways that are developmentally and immune associated are differentially regulated as compared to a control larva, such as the Hedgehog, Wnt, and JAK/STAT pathways. Several proteins of interest in immune function and wound healing are also observed to be specific to the anti-nematode response, which includes the involvement of a complement-like protein, TEP3, a basement membrane component, glutactin, and a recognition protein, GNBP-like 3 [13,14]. Nematode infection has also been determined to be inhibited by several proteins that are clot-associated, such as transglutaminase, IDGF2 and 3, and fondue (see FlyBase.org for more gene details [15,16,17]).

While details of *Heterorhabditis* and *Steinernema* infection in *Drosophila melanogaster* have been explored in several studies, including important humoral and cellular components of host immunity, the exact mechanism of entry, subsequent proliferation in early stages of infection, as well as the determination of an advanced point of infection have not yet been fully elucidated. In this study, we used high-resolution microscopy to gain further knowledge on the mode of entry and proliferative events of *Heterorhabditis bacteriophora* and *Photorhabdus luminescens*. We also distinguished early infection from advanced infection at a time point at which bacterial sepsis overcomes the host and eventually leads to host mortality. We found that EPNs enter through the cuticle of the host with a twisting action, resembling the mode of entry of other parasites, as well as determined the timing of septicemia, loss of tissue integrity, and specific nematode titers, which will lead to septic events. With these data, we provide increased temporal and spatial infection kinetics, which can further increase our knowledge regarding a threshold for overcoming the host immune system, which has implications for both the agricultural industry and human health.

## 2. Materials and Methods

### 2.1. Fly Strains and Handling

The fly strain w^1118^ was obtained from the Bloomington Drosophila Stock Centre. Flies were maintained under a 12-h light/dark cycle and fed with a potato agar mash (12.9 g of dry yeast (Kron Jäst; Sollentuna, Sweden), 40 g of potato powder (Felix Potatismos; Skåne, Sweden), 10 g of agar (USBiological; Salem, MA USA), 50 mL of light syrup (Dansukker; Malmö, Sweden), 8.5 mL of nipagin (10% in 99% Ethanol, Sigma-Aldrich), 1 g of L-ascorbic acid sodium salt (AlfaAesar; Kandel, Germany), 4.5 mL of propionic acid, 99% pure (Acrōs Organics; Fisher Scientific), and water up to 1 L). Adult flies were primed with excess yeast for 2 days at 25 °C, allowed to lay eggs for six hours, and then placed at 29 °C. Hatched 1st instar larvae were transferred to vials of 3 mL of standard food to control for crowding and placed back at 29 °C. Larvae were either washed out with tap water or selected individually from the food mash 72 to 76 h after egg deposition (AED). In order to determine gut integrity, larvae were fed with colored food. If a loss of tissue integrity occurred, color entered the open circulatory system of the larva and were subsequently dubbed “Smurfs”. For Smurf Assay experiments, selected larvae were allowed to feed for 2 additional hours on food containing pH indicators Bromothymol Blue, Thymol Blue, Congo Red, Bromocresol Purple, or Crystal Violet Solution [18,19,20].

### 2.2. Nematode Culturing

*Heterorhabditis bacteriophora*, which harbor the symbiotic bacteria *Photorhabdus luminescens* TT01, were cultured and used throughout all experiments. EPNs were cultured in the Greater Wax Moth, *Galleria mellonella* at room temperature. Infective juveniles (IJs) of EPNs were maintained in tap water in vented culturing flasks. EPNs were diluted in tap water to a density of 50 IJ/10 μL for both species. The age of the nematodes ranged between 21 and 50 days after the emergence from the hosts’ cadaver.

### 2.3. Infection Assays in Drosophila

The infection assay was adopted from Dobes et al. [21]. Fifty L3 larvae were placed in a re-sealable transparent plastic bag (6 × 10 cm), in which one layer of a 6 × 10 piece of Whatman paper was soaked with either 50 or 500 IJs/larva depending on the assay or to different titers of nematode suspension to determine the effective dose at which 50% of the larvae had become septic after 6 h, or multiplicity of infection (MOI). Titers were tested in triplicate and included: 0, 2, 5, 10, 25, 50, 75, 100, 250, and 500 IJs/per larva. Across all titers and water controls, the volume of water stayed constant so as not to oversoak the bag (250 μL/bag). Infections were conducted at 29 °C in the dark. Septicemia was quantified after 0, 2, 4, 6, and 8 h, and was scored positive when bacteria were spread throughout the larval cavity. Green Fluorescent Protein-expressing *Photorhabdus* (a kind gift from Todd Ciche, referred to in the text as Ht-GFP) were used to monitor the spread of infection in the larvae. All experiments were run in triplicate using 50 larvae per bag.

### 2.4. Imaging and Microscopy

Larvae for time-lapse microscopy were incubated with 500IJs/larva for 30 to 45 min to select for larvae that were in the early stages of nematode attack and entry. L3 *Drosophila* larvae were adhered to a 13-mm round 1.0 coverslip using the glue method, or a thin layer of superglue [22]. After 30 s of drying, the glued larva was inverted and placed onto a 35-mm dish, No. 1.5 Coverslip, 14-mm glass diameter, Poly-d-lysine Coated (MatTek). Ringer’s Solution was added to the periphery of the petri dish to keep the atmosphere humid and larval body moist. Confocal imaging was conducted through a Zeiss Fluar 5×/0.25 objective attached to an inverted LSM 800 Airyscan microscope (Carl Zeiss, Jena Germany), which was controlled by the ZEN blue 2.1 software. The latter software was also used to select and enhance the slices and create maximum intensity projections of Z-stacks and time-lapse series. The Smurf assay larvae were adhered to a glass slide using double-sided tape. They were subsequently imaged with a DFC 300Fx digital camera using Firecam imaging software (version 3.4.1) through an MZ16 Stereomicroscope (Leica, Wetzlar, Germany) set to 1.6× magnification. Ht-GFP-infected *Drosophila* were counted using an Axioplan2 light and UV microscope (Carl Zeiss, Jena, Germany) with a 4× objective and a Hamamatsu ORCA digital camera controlled by Axio Vision Rel.4.8 software.

### 2.5. Larval Behavioral Tracking Using FIM Software

The behavior of the infected larvae was determined according to the protocol in Kunc et al. [11]. In short, 0.8% agarose gel with a 2-mm thickness was used as a crawling surface, and a salt barrier was poured to prevent larvae from escaping the experimental area by adding 5M NaCl in 2.5% agarose gel in deionized water. Larvae were starved for 2 h before the start of the experiments and 10 replicates, which contained 10 larvae per run, were analyzed per condition for each experiment. The size of the images was 1000 × 1000 pixels and captured with a frequency of 1 FPS (frame per second) for 720 s. The scale factor was 100 pixels/cm. A Basler A601f camera coupled with FIMTrack v2.1 Windows (X86) was used [23]. All larval locomotion tracks were initially recorded and processed with the software and then manually verified.

### 2.6. Statistical Analyses

Statistical analysis and graphs were assessed and produced using GraphPad Prism software, version 8.0. Z-stacks of selected images were exported to IMARIS v. 9.5 software (Bitplane AG, Zurich, Switzerland) and made into 3D movies. All experiments were performed a minimum of three times. The results are expressed as the mean ± SD; the level of significance was analyzed either by Chi-square tests, a Mann–Whitney test, or an unpaired t-test depending on the D’Agostino and Pearson test for normality. The multiplicity of infection, described above, was measured in Prism using a non-linear regression called “[Agonist] vs. response—Variable slope” after log transformation and normalization of the data. The nematode represented the agonist and the response reflected the percentage of larvae which had become septic. Data gathered from confocal images were processed and visualized in Prism or IMARIS.

## 3. Results

### 3.1. Mode of Entry of Heterorhabditis into the Drosophila Cuticle

Entomopathogenic nematodes (EPNs) are known to enter the host either through the cuticle or through open orifices, with certain species of EPNs preferring one mode or both modes of entry. In the case of *Heterorhabditis bacteriophora*, it has been documented to enter through the mouth and anal cavity as well as by penetrating the cuticle [14]. However, the exact mechanism of entry through the cuticle and exsheathment has not yet been observed for *Heterorhabditis bacteriophora*. In order to follow the EPN *H. bacteriophora* in its effort to gain entry into the hemocoel of *Drosophila* larvae, we used a combination of *H. bacteriophora* and its symbiotic counterpart, *Photorhabdus luminescens*, which contained a GFP-expressing plasmid [1]. We exposed w^1118^ larvae to high titers of EPNs, 500 IJs/10 μL/larva, for 30 to 45 min until larvae were swarmed with many EPNs; EPNs preferred some individual larvae compared to other nearby larvae. A larva that had not yet been infected but was swarmed, as determined by an absence of a GFP signal within the cavity of the larva, was selected, unwashed, and carefully placed onto a 13-mm cover glass with superglue. Time-lapse microscopy was subsequently used to observe EPN behavior upon penetrating the host.

**Figure 1:**
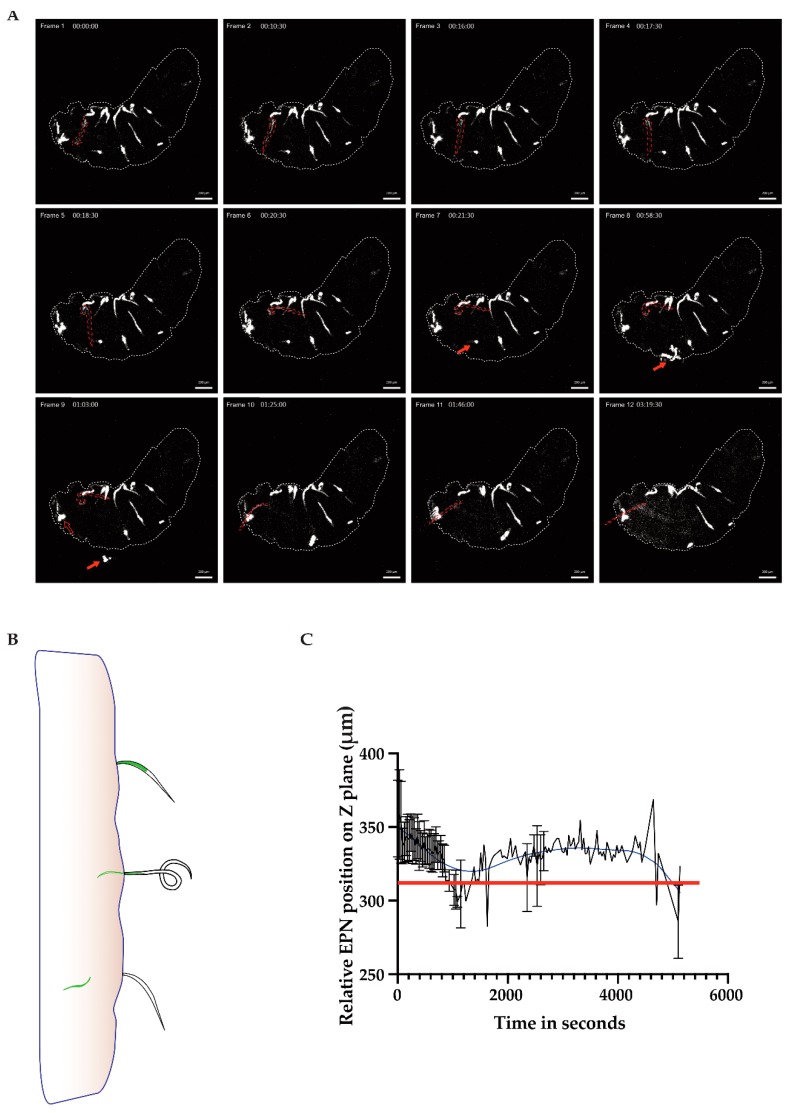
Time-lapse series of *Heterorhabditis bacteriophora* entering the *Drosophila* cuticle. L3 w^1118^ larvae were infected with 500 IJ/ 10 μL per larva in a bag assay. After around 45 min, a larva was selected, glued to a 13-mm coverslip, and imaged using a confocal microscope, the LSM 800. Time lapses were taken for 5 to 6 h documenting the early stages of infection in *Drosophila*. Stills demonstrating key points of interest from a representative larva being infected with an entomopathogenic PN, outlined in red, are shown, scale 200 μm (**A**). The filled arrows in frames 7–9 show an EPN detaching. The open arrow in frame 9 shows the anterior region of an EPN partially inside the cuticle but remains unable to enter throughout the video. A schematic of EPN entry into the cuticle (**B**). A graph showing the spatial location of the EPN of interest over time. GFP intensity is followed over time relative to the position in the Z plane or the spatial relation of the inner EPN cavity being below or above the cuticle, indicated with a red line on the graph; the blue line indicates the smoothed trend (**C**). When the position of the EPN drops below the red line, it has breached the cuticle barrier (see Movie S2). This figure is a representative video of entry based on observation (see text for more details).

In Figure 1, we saw several EPNs adhered themselves to the cuticular surface of the larva; however, only one would go on to enter the larva (frame 1, nematode of interest outlined in red). The nematode of interest engaged in a twisting motion in order to position itself for entry (frame 2–6), and after about 45 min, it finally disengaged its inner from its outer cavity (frame 9–10), leaving behind an empty sheath that stayed appended to the outside of the host cuticle. The IJ then entered the host and started to regurgitate bacteria from the point of origin (frame 10–12; Figure 1A time series, outlined larva; see also Movie S1, a time-lapse and 2, a 3D reconstruction of the entry moment with a focus on the nematode of interest). Although this larva had around 16 EPNs attached to the cuticle, only one successfully entered the cuticle while another EPN, pictured in frame 8 (red filled arrow), maneuvered itself violently enough until it is seen detaching in frame 9 (see arrows). Another EPN (the empty red arrow) is seen throughout the time-lapse partially inserted into the host cuticle while never successfully gaining entry.

Of note in Frame 12 in Figure 1A is the bacterial dissemination from close to the point of entry towards the anterior end of the larva—rather than being taken up into the larva’s open circulatory system, the bacteria appear to attach to the walls of the organ and spread towards the anterior end of the larva. These data support previous evidence for a functional compartmentalization of the hemolymph upon injury [24]. A schematic of the point of entry shows the nematode at first attached to the host’s cuticle; then it dislodged its inner cavity from its outer sheath, which allowed it to finally enter the host with its symbiotic GFP-expressing bacteria (Figure 1B). Using the image processing software, IMARIS, the nematode of interest was tracked according to its GFP pixel intensity and spatial relation (z plane coordinates) in order to determine if the EPN was either above or below the cuticular barrier of the host (red line, Figure 1C; based on Movie S2). The GFP signal concentrated from the middle of the EPN to the tip of the barrier between the EPN and the larva (shown with the red line on the graph); the signal descended through z positioning over time as the inner cavity of the EPN entered the inner side of the host.

The drill-like motion for entry into the host may demonstrate convergent evolution with other parasite species, such as *Toxoplasma gondii* [25]. The speed of entry of the outlined EPN and the low success of entry of other EPNs in these frames could be the result of the larva being immobilized to the cover glass, rendering the infection scenario not ideal for the nematodes; thus, a certain threshold of entry was needed in order to finish the entry process. Once exsheathment occurs, which exists to protect the worm from harsh environments, the infection process can be carried out [26].

### 3.2. Tracking EPN Trajectory within the Host

**Figure 2:**
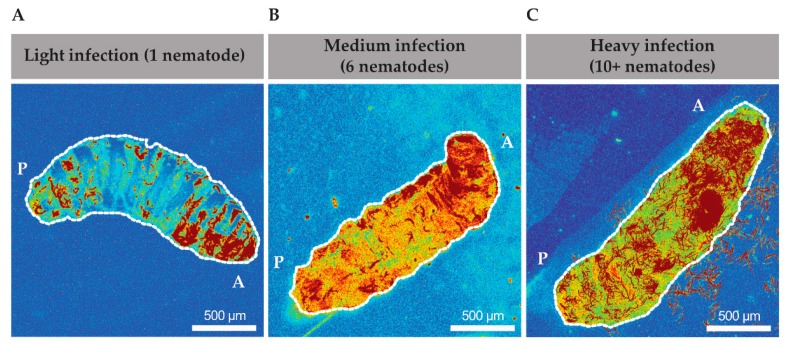
Heat maps of EPN *Heterorhabditis bacteriophora* movement inside of the host, *Drosophila melanogaster*. Time-lapse images were processed into maximum intensity projections of individual time points which were then combined throughout acquisition. Representative images are taken for low (1–4 EPNs, **A**), medium (5–10 EPNs, **B**), and high (10+ EPNs, **C**). “A” marks the anterior side of each larva while “P” marks the posterior side of each larva. Scale bar—500 μm.

Some parasites require specific tissues or environments within the host in order to differentiate into different stages of their life cycle, as is the case with *Plasmodium falciparum* travelling to hepatocytes to go from a sporozoite to a merozoite [27]. As EPNs will differentiate into the sexual stage after about 24 h, we wondered if there was a region where EPNs would be most likely to be found within the host cavity once an infection occurred. In order to determine if there was a predictable behavior of nematodes within the host cavity, we tracked *Heterorhabditis* via their GFP-expressing symbionts and created heat maps of time lapses that were taken from 1 to 6 h after infection. We found that EPNs seemed to spend a majority of time in the anterior end of the larvae (Figure 2A–C). In a low infection scenario, 1 to 4 EPNs, the pixel intensity was demonstrated to be the highest in the anterior end of the animal (Figure 2A). In a medium infection scenario, with 5 to 10 EPNs, a more distinct track was found to be travelling along the dorsal side of the animal from posterior to anterior and similarly showed a higher pixel intensity of GFP at the anterior end (Figure 2B). Finally, in a high infection scenario, with greater than 10 nematodes, a very distinct track is found along the dorsal end towards the anterior side (Figure 2C).

Thus, even across different infection intensities, nematodes behaved similarly across different infection loads and migrated towards the anterior region primarily. In several high infection scenarios (Figure 2C and data not shown), nematodes were seen to move from within the body out through the oral cavity and occasionally transgressed through the oral cavity in both directions. Therefore, the movement of nematodes within the cavity did not appear to be random. Further exploration and understanding of the infection biology are necessary to determine if this behavior has a role in the EPN’s ability to overcome the host’s immunity and subsequently differentiate into the sexual stage.

### 3.3. Rate of Bacteria Proliferation after EPN Infection

**Figure 3:**
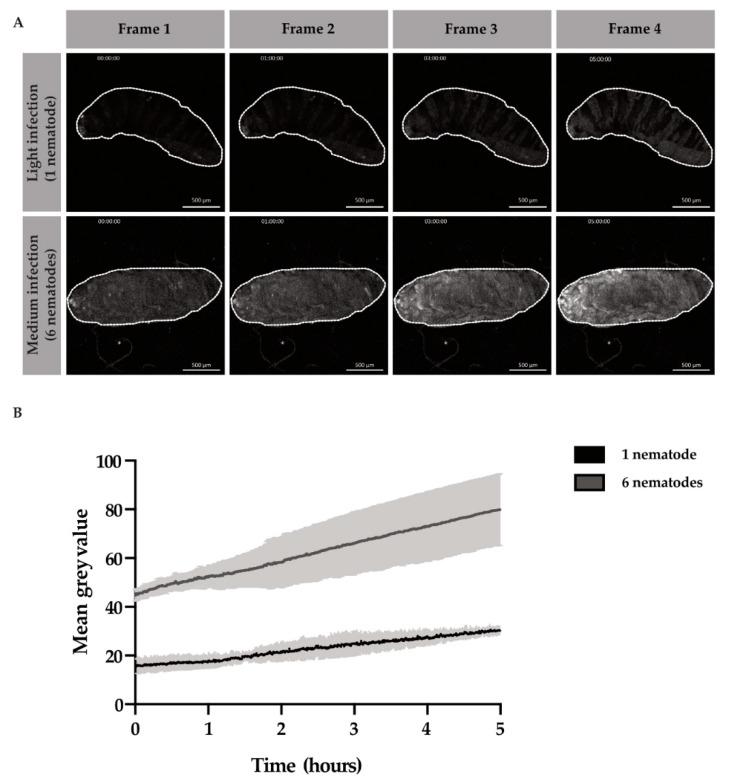
Light and medium infection of the EPN *Heterorhabditis bacteriophora* and their symbiotic bacteria, *Photorhabdus luminescens*-GFP, in *Drosophila melanogaster*. Time-lapse images acquired for light and medium infection that show stills from 0, 1, 3, and 5 h after 1 h of infection (**A**). Graphs demonstrating the mean grey value of bacterial proliferation of *Photorhabdus luminescens* inside the larva from 1 to 6 h after infection (**B**). Scale bar 500 μm.

After entry into the host hemocoel, EPNs regurgitate their bacteria, which will subsequently proliferate in the host cavity. However, it is unknown how rapidly the nematodes regurgitate their gut bacteria, *Photorhabdus luminescens,* and how long subsequent bacterial proliferation takes to cover the host cavity and cause sepsis. We also wondered how different titers of nematodes could affect the rate of bacterial proliferation. We observed in Movie S1 that after entry into the host, bacteria were released close to the point of entry and progressed throughout the larval cavity from the posterior to anterior end in about 6 h. Thus, we decided to compare the proliferative events of the bacteria during the first 6 h of infection. We infected larvae and selected for either “light,” infection loads of 1 to 4 EPNS or “medium” infection loads of 5 to 10 EPNs. After larvae were infected with the EPN(s) for 1 h, time-lapses commenced. Bacterial GFP pixel intensities were compared at “t0”, or 1 h after infection, until “t5”, or 6 h after infection. Across light and medium infection, the GFP intensity, or mean grey value, increased over time (Figure 3A). We found that bacterial proliferation was three times higher in the six-nematodes infection scenario than the one-nematode infection scenario after 5 h of time-lapse imaging (Figure 3B).

The number of EPNs inoculating the larva with bacteria sped up the infection process by 3 times. Furthermore, the immune responses of the larvae may vary depending on the inoculation load of the larva. With this data, we determined a window for the infection period in which the immune response likely gets mounted and subsequently overcome. These are important data to consider when comparing individual larval immune responses to one another in genetic studies; different rates of bacterial proliferation will likely lead to varying rates of sepsis as well as immune induction responses.

### 3.4. Sepsis and Multiplicity of Infection

**Figure 4:**
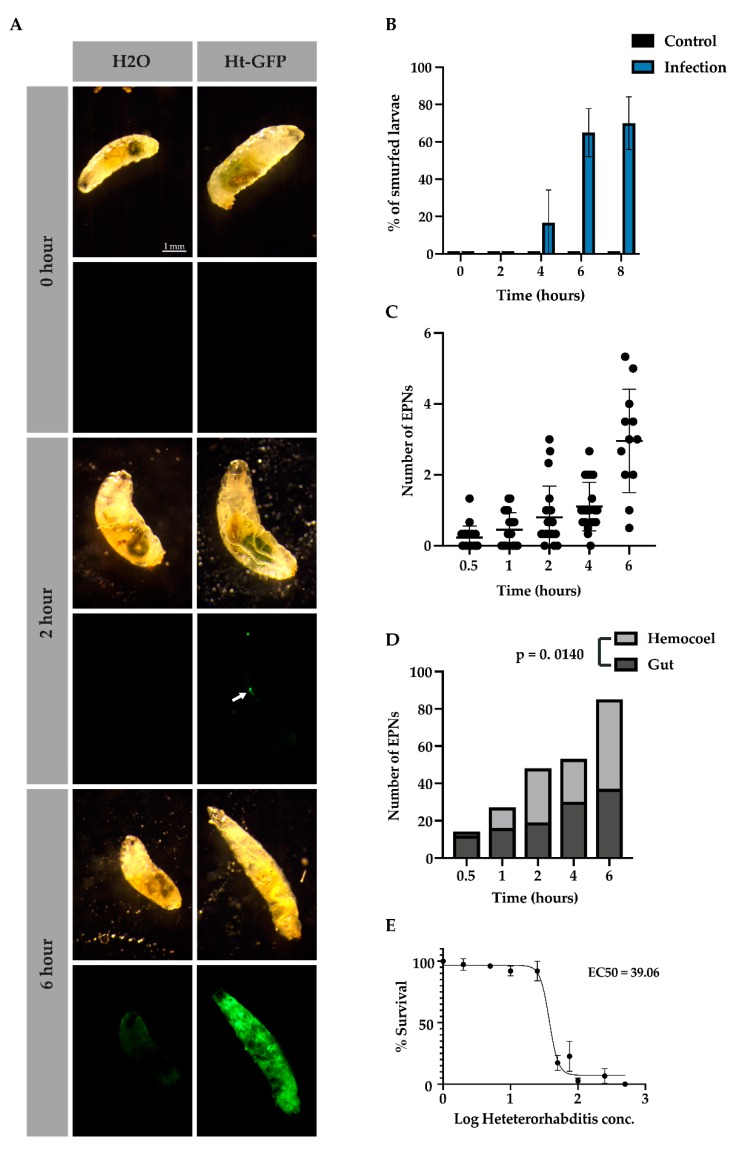
Nematode infectivity and multiplicity of infection in septic animals. Representative images of larvae that were infected with *Heterorhabditis* (Ht-GFP) or in the control (H_2_O) condition over 0, 2, and 6 h (**A**), scale bars indicate 1 mm. Arrow at the 2-h time point demonstrates a single nematode inside of the host. Infected larvae (Ht-GFP) and control larvae (H_2_O) were scored after 0, 2, 4, 6, and 8 h of EPN exposure to determine the percentage of larvae that had lost tissue integrity and become septic (**B**). The average number of EPNs infecting a single larva over time (**C**). Infected larvae were scored based on whether EPNs were found inside the gut or the host hemocoel, Χ^2^ (12.5, N = 4), *p* = 0.0140 (**D**). The multiplicity of infection for *Heterorhabditis* was determined using the GFP-expressing symbiotic bacteria, *Photorhabdus luminescens*. in total, 50% of the population were determined to be septic, in which bacteria were found throughout the host cavity, and an effective concentration of 39.09 EPNs per 10 μL per larva was found (**E**). All experiments were performed in triplicate.

As we observed that bacteria were able to cover the host cavity within 6 h in Figure 1 and Figure 2, we hypothesized that a loss of tissue integrity occurred in this time, which would lead to sepsis within the host. To determine the loss of gut integrity, we fed larvae for 2 h on 4 different pH dyes. The pH dyes covered ranges across the whole pH scale to determine if virulence factors secreted from bacteria used changing ion concentrations to create a more favorable environment for replication and eventually host death. We used 4 pH indicators as well as a regular cell stain, Crystal Violet Solution, to visualize the dye better and to control against indicators causing the tissue lysis (Figures S1 and S2). When color was observed in the open circulatory system of the larva, the larva was dubbed a Smurf, which in our scenario represented septicemia [18,20]. We found that EPNs did not affect gut pH when releasing virulence factors (data not shown) and that gut integrity, as detected with bromothymol blue, remained intact after 2 h, started to deteriorate by 4 h, and had largely deteriorated by 6 and 8 h (Figure 4A,B). In Figure 4A, at the 2-h time point, an EPN was seen inside the larva that had not yet released its symbiotic bacteria (arrow) while at the 6-h time point, sepsis was observed after the bromothymol blue turned from blue to yellow (hemolymph has a pH of 7, see Figure S1 for pH details).

Since *Drosophila* larvae are differentially attractive to EPNs based on olfactory cues and other unknown factors [12], we sought to determine how many larvae in a pool were infected by how many EPNs. We found that over time, at an infection rate of 50 IJ/10 μL per larva, EPN numbers increased inside the host from 1 EPN at 1 h to about five EPNs at 6 h (Figure 4C). We further wondered whether EPNs were more likely to infect *D. melanogaster* either through open cavities or through the cuticle. Interestingly, EPNs were more likely to be found in the gut in early infection; however, over time, they were more significantly found within the host hemocoel (Figure 4D, Χ^2^ (12.5, N = 4), *p* = 0.0140). This effect may be explained by the loss of tissue integrity at later time points but may indicate a preference at early time points. Since distinct levels of nematode infection varied over time, which affected the rate of bacterial proliferation and thus sepsis for each individual, we then wondered if there was a titer of nematodes that will usually lead to sepsis within 6 h. We found that, when infecting *Drosophila* larvae, an effective dose of about 40 IJ/ 10 μL per larva led to at least 50% of the larval population being septic after 6 h (Figure 4E).

While no change in pH was detected based on bacterial proliferation, though another method may be more sensitive, infected larvae were observed to lose tissue integrity by 6 h of infection. Together, these data indicate that there is an effective dose and time by which EPNs are likely to have overcome the host’s immune system. Also of note, is that the wounds that the EPNs inflict on the host do not cease once it has entered through the gut or the cuticle. Loss of tissue integrity continues throughout the infection, which likely leads to the activation of additional wounding and clotting factors.

### 3.5. Larval Behavior upon Early Infection

The question arose while observing septic larvae of whether or not they showed signs of lethargy or mortality, due to their lack of movement in later stages. We observed that larvae that were septic were still alive; however, they exhibited lethargy and malaise (Movie S3). Of note in Movie S3 is that an EPN can be seen exiting through the mouth during the time-lapse while the larva slowly moves its anterior end towards the left side of the frame. Thus, sickness behavior was observed in septic larvae (Movie S3) and Smurf larvae (Figure S2). Those that were infected showed lethargy and decreased gut peristalsis and function, evidenced through the spreading of the dye throughout the larva after several hours as opposed to healthy larvae, which mostly lost gut coloring due to bowel function after the same incubation (Figure 4). It could be desirable to decrease normal organ function in order to allocate additional resources towards mounting an immune response and overcoming infection [28].

**Figure 5:**
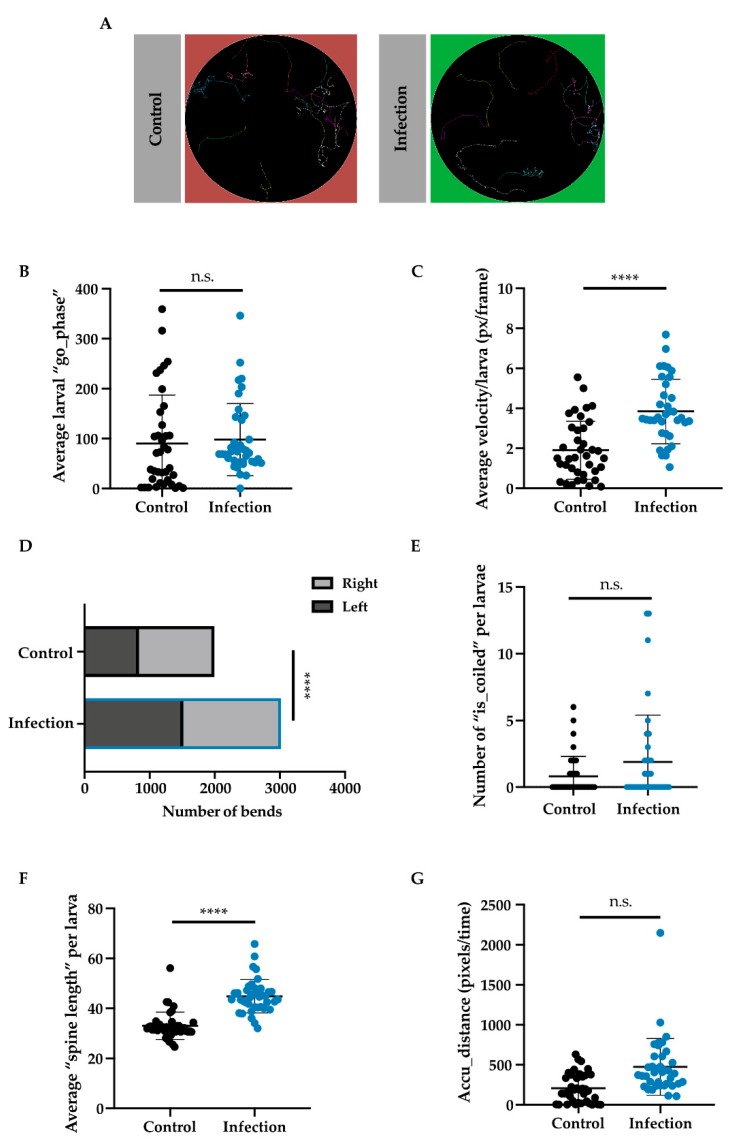
Tracking larval behavior after infection with *Heterorhabditis*. Over 480 s, larval behavior was tracked using Frustrated Total Internal Reflection-Based Imaging Method (FIM) software. Representative images of larval tracks (**A**). One still was taken per second, and the larval movement was detected using parameters, such as the go phase (**B**), velocity (**C**), bending preference (**D**), coiled (**E**) spine length (**F**), and accumulated distance (**G**). Each dot represents a larva’s average value in that parameter throughout the imagining. Forty larvae were analyzed, and data are shown as mean ± SD; n.s. = not significant, **** *p* < 0.0001.

We then began to wonder how infection may affect other larval behaviors, such as locomotion, at earlier stages of infection. In order to test this, we used an infrared table coupled with FIM tracking software designed to follow larvae [23]. Larvae were infected with Ht-GFP EPNs and then selected for tracking. Representative images of larval tracks showed similar tracks between infected and non-infected larvae (Figure 5A). The go phase (or a positive score for movement between two stills) and velocity (pixels/frames per second) were both plotted and checked for significance. While larvae did not appear to differ in the amount they moved (Mann–Whitney U = 599.5, n_1_ = n_2_ = 38, *p* = 0.2053 two-tailed), there was a significant difference between control larvae (M = 1.907, S.D. = 1.450) and infected larvae (M = 3.843, S.D. = 1.615) regarding their velocities (t (72) = 5, *p* < 0.0001 two-tailed; Figure 5B,C). Bending and coiling behavior were examined and while coiling (or head to tail touching) did not differ between groups (Mann–Whitney U = 637, n_1_ = n_2_ = 38, *p* = 0.3148 two-tailed; Figure 5E), larvae that were infected bent themselves much more than control larvae (Χ^2^ (33.17, N = 38), *p* < 0.0001; Figure 5D). Further, spine length (or the length of the larva in pixels) was found to be longer in infected larvae (M = 44.86, S.D. = 6.69) as compared to control larvae (M = 33.04, S.D. = 5.47); t (76) = 8.547, *p* < 0.0001 (Figure 5F). Larvae were also analyzed based on their accumulated distance (or the number of pixels they travelled over time) and we found that infected larvae travelled further than control larvae (Mann–Whitney U = 298, n_1_ = n_2_ = 38, *p* < 0.0001, two-tailed; Figure 5G).

Thus, larvae in early infection scenarios showed significantly different behaviors in terms of velocity, bending, and distance travelled but not for movement, coiling, or spine length. While control and infected larvae may have moved equally in the go phase, infected larval tracks appeared to be more zigzagged, which could correspond with their increased bending and spine length. Rather than showing sickness behavior, as observed in septic larvae, the early behavioral response to infection seemed to lead to greater energy expenditure. Perhaps the behavior from early infected larvae demonstrates the individual’s last-ditch effort to rid itself of infection, or perhaps it helped the larva initiate a strong immune defense—further research is necessary to elucidate whether or not there are any advantages or disadvantages conferred by these behaviors.

## 4. Discussion

Nematodes and their symbiotic bacteria represent a complex infection system. In our study, we focused on the early infection wounding, entry, and proliferation events that have been known but have evaded inspection at this level of high resolution and detail. We found that the EPN, *Heterorhabditis bacteriophora*, enters through the cuticle with a drill-like motion, twisting around its focal anchorage point to dislodge the inner cavity from the outer sheath. While this process has been observed to occur at higher frequencies under a 1-h, in our scenario, the EPN entered after about 1.5 h. This may perhaps indicate the importance of the EPN having a definitive surface to push off from to enter the host. The sheath subsequently stayed attached to the outside while the EPN started to regurgitate bacteria within the first hour of entry. After about 6 h, the symbiotic bacteria, *Photorhabdus luminescens*, was detected throughout the entire cavity of the larva. Unsurprisingly, the speed of bacterial proliferation increased with higher numbers of EPNs, leading more rapidly to septicemia, likely aided by the open circulatory system of the larva and combined inoculation from multiple nematodes. Subsequently, signs of swelling or edema were observed. Finally, EPNs were observed to freely exit and on occasion re-enter the host through the mouth after infection had occurred.

EPNs were observed to selectively swarm a host prior to infection compared to nearby larvae that were free of EPNs. A swarming behavior may be evolutionarily advantageous for taking down a single individual host but may also reflect unknown olfactory or fitness cues that make a single host more attractive than another [12]. After wounding the larva and gaining entry, frequently through the gut in earlier time points while through the cuticle in later time points, septicemia occurred in early stages of infection regardless of the number of nematodes that entered. While septic, larvae remained alive and continued to fight the infection; thereafter, they were overwhelmed by tissue degradation and virulence factors, eventually leading to mortality. While it is unknown if any host behavior is directly beneficial to the EPN while inside, it is possible that the behavior of an infected individual likely sends cues to surrounding larvae about the danger of pathogenic threats in the area [29,30]. Furthermore, infected larvae exhibited traits of known sickness behaviors, which included malaise, lethargy, and reduced bowel movements, as evidenced through the dye maintained in their guts for several hours longer than the control. Such sickness behavior presumably shifts metabolic costs from motility and feeding to an energetically intensive immune response [28,31,32].

Through the use of the glue method for larval immobilization in conjunction with high-resolution microscopy, we developed a method for gaining a clear and accurate understanding of the early infection kinetics of EPNs. These combined methods can help bring clarity to the host’s immune response as well. With the array of genetics tools available to *Drosophila*, many reporter lines and fluorescently tagged proteins can be followed in real-time in response to EPN infection or other infectious agents. Furthermore, several factors have been identified in the *Drosophila* host response against nematodes [13,15]. Testing, for example, the rate of bacterial proliferation and success of infection using knockdowns and mutant strains will be of great interest for the field.

Furthering our understanding of this complex tripartite system is of particular importance in the context of wound healing. Wound- and clot-associated proteins have been reported to have pleiotropic effects regarding traditional wound healing, protection against nematode entry, as well as an immunological function against the nematode once the EPN has breached the barrier [15], possibly through the dual roles of extracellular matrix components and crosslinking proteins, which act in conjunction to inhibit the EPN’s entry. Other clotting and immune genes, which include *IDGF3* and the complement-like *TEP3* genes, have an anti-nematode role, which may make EPN infection akin to a murine type-II immune response [14,33,34]. One clotting gene that is most conserved across all organisms is transglutaminase, or factor XIIIa in humans, and has been found to have an anti-nematode effect in *Drosophila* [16]. In response, EPNs have developed several strategies to overcome immunity and have been reported to secrete peptidases and inhibitors that have specific activity against prophenoloxidases and other clotting genes [35,36]. Other nematode species have developed a protective mechanism against reactive oxygen species from the host [37]. Interestingly, nematodes require transglutaminase to catalyze reactions for development, body maintenance, and morphology [38]. Whether or not transglutaminase is needed to invade the host and bind to the cuticular barrier is currently unknown. This evolutionary arms race is expected according to the red queen hypothesis, which posits that hosts and parasites evolve more distinct and specific responses towards one another, ultimately driving their coevolution [2,39]. Thus, with such a specific species interaction, EPNs present themselves as a model system in *Drosophila* for furthering our understanding of host–parasite interactions. Not only can this system help us to learn more about nematode infections, but it can also bring forth greater clarity concerning wounding, edema, septicemia, and other advanced inflammatory responses [40].

## 5. Conclusions

Infectious nematodes are an important model in understanding the insect response to injury and activation of the immune system. In our study, we found that infectious nematodes enter through the host’s cuticle in a drill-like fashion. Symbiotic bacteria were immediately released after entry, and after 6 h, they had spread through the entire host cavity. Degradation of the host tissue and overcoming the host immune response appeared to be time dependent, usually occurring within 6 h. The host was able to sustain septic levels of bacterial infection for about 2 h before succumbing to the infection load. Further, we found that after 6 h, 50% of larvae were likely to have become septic at the effective concentration of 40 infectious juveniles per 10 μL of EPNs per larva. Thus, our study provides high spatiotemporal resolution of the early infection kinetics in *Heterorhabditis bacteriophora* which better elucidate the likelihood of infection and infection events for each larval host in *Drosophila melanogaster*. With this insight, we can better understand when to target the host’s anti-nematode response to further our understanding of clotting factors, immune factors, or hemocyte behavior [4,41].

## Supplementary Materials

The following are available online at https://www.mdpi.com/2075-4450/11/1/60/s1, Movie S1: An 18-h time-lapse video of a L3 *Drosophila* larva that is being attacked by many nematodes which are of the species *Heterorhabditis bacteriophora*, containing symbiotic gut bacteria, *Photorhabdus luminescens* with a GFP-expressing plasmid, Movie S2: An IMARIS 3D reconstruction video of the point of entry of the nematode in Figure 1 and Movie S1. The GFP signal is indicated in green with the volume following the spatial location of the bacterial GFP pixel intensity over time. The z-plane presented in the video represents the cuticular surface of the larva Movie S3: A septic larva that was imaged over 6 h to determine whether lack of movement was due to lethargy or mortality. A larva was placed on a Petri dish with no glue. A nematode is seen escaping through the mouth of the larva after 2.5 h, Figure S1: Four different pH indicators were used to assess alteration in gut pH upon EPN infection. pH indicators are depicted in the early L3 larvae as well as in their dissected guts after 2 h of feeding with no infection (**A**). Each indicator is described concerning which color it changes to at a specific pH range (**B**). (Scale bars indicate 0.5 mm and 1 mm for larva and gut, respectively), Figure S2: Scoring of “Smurf” larvae with Crystal Violet Solution. After 6 h of infection, several smurfing phenotypes could be observed likely due to the variability of infection time. In the left-hand panel, high infection loads can be observed while guts remained intact, while in the right-hand panel, loss of gut tissue integrity was detected (**A**). All experiments in the article were conducted in the modified bag assay depicted, using a single layer of Whatman paper for its absorbency (**B**). Scale bar indicates 0.5 mm.
