# Supplementary material for: High-Resolution Infection Kinetics of Entomopathogenic Nematodes Entering Drosophila melanogaster
Source: Insects. 2020 Jan 18;11(1):60. doi: 10.3390/insects11010060 (PMC7023307; doi:10.3390/insects11010060)
Supplement: Supplementary file 1 [file insects-11-00060-s001.zip › Insects-2-sup/Supplementary movie legends.docx]

Movie S1: Entry and Infection time-lapse video. A nematode enters the larva after 1.5 hours and starts to regurgitate symbiotic bacteria. The video is about 18 hours of time-lapse with frames taken 30 seconds apart (See Figure 1 time-lapse stills and text for more details). Time-lapse was taken on an LSM 800 confocal microscope. Scale bar 500 μm.

Movie S2: 3D reconstruction of entry and infection of a nematode from Figure 1. Movie was formatted in IMARIS. Movie shows 1 hours and 40 minutes of the entry process. Scale bar defined over video.

Movie S3: Septic Larva showing sickness behaviour. After 4-6 hours of infection, a larva showing sepsis was followed for about 4 hours with frames taken 30 seconds apart. Time-lapse was taken on an LSM 800 confocal microscope. Scale bar 500 μm.
