# Supplementary figures and images for: High-Resolution Infection Kinetics of Entomopathogenic Nematodes Entering Drosophila melanogaster
Source: Insects. 2020 Jan 18;11(1):60. doi: 10.3390/insects11010060 (PMC7023307; doi:10.3390/insects11010060)

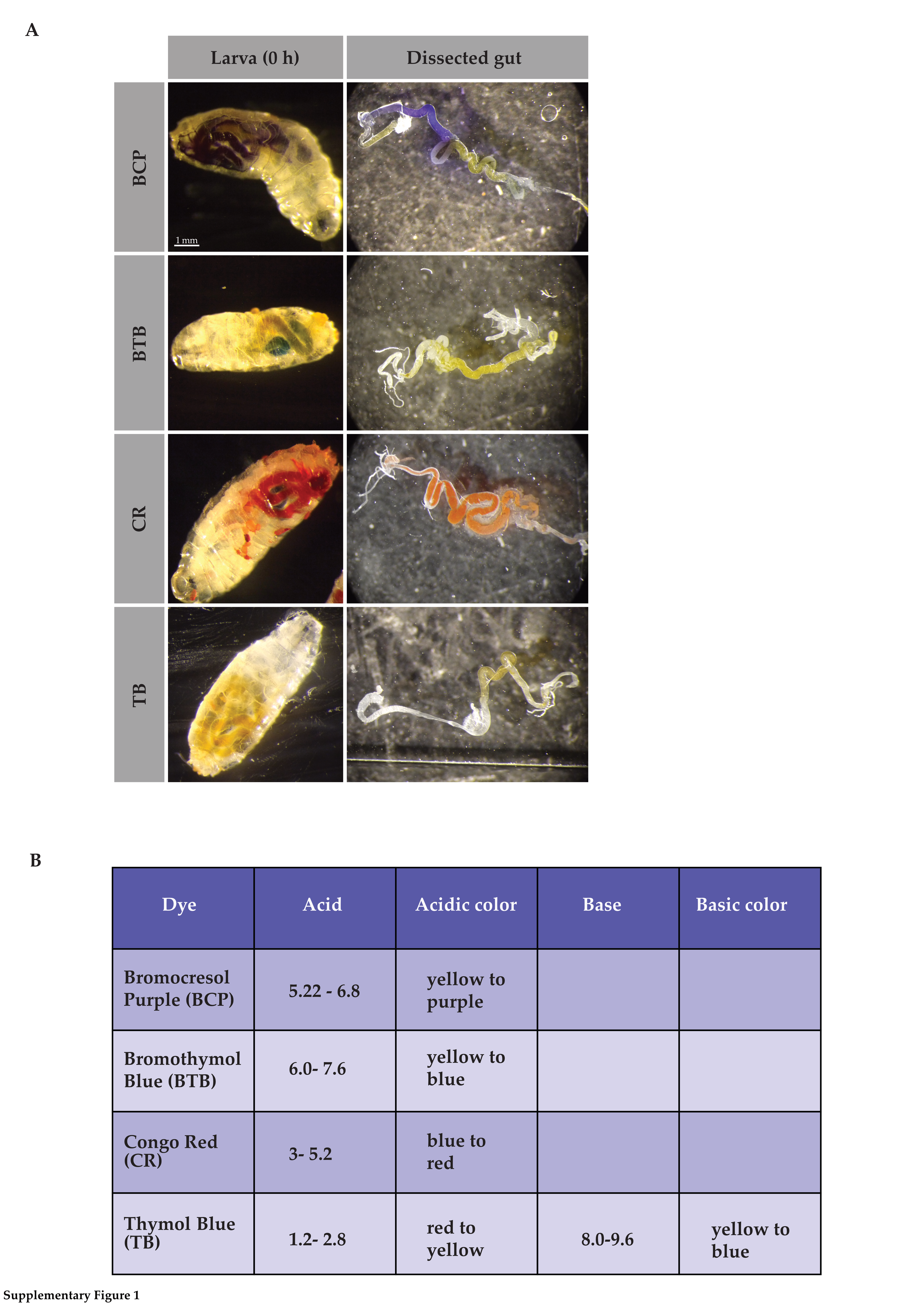

Supplement: Supplementary file 1 [file insects-11-00060-s001.zip › Insects-2-sup/Supplementary figure 1.png]

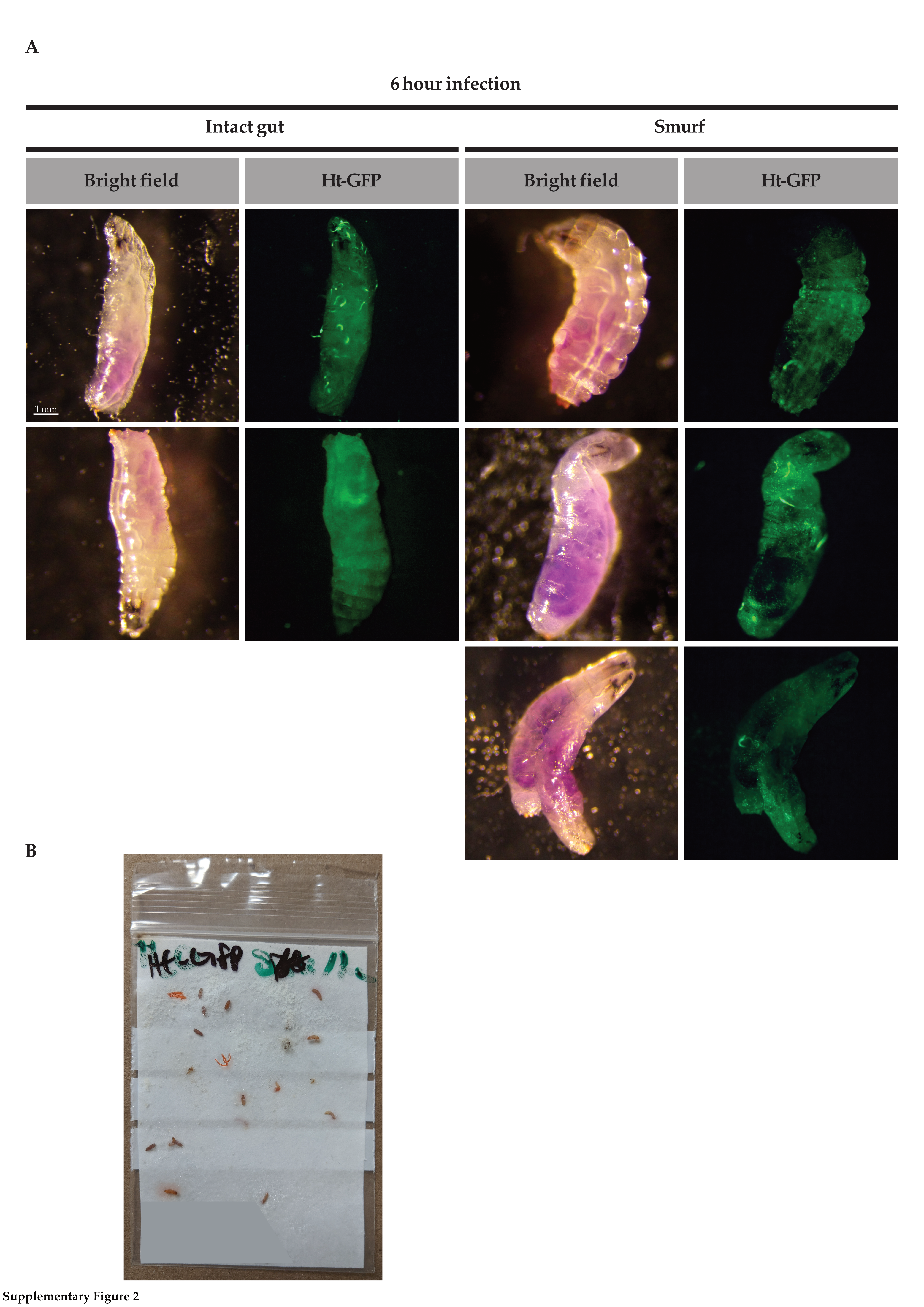

Supplement: Supplementary file 1 [file insects-11-00060-s001.zip › Insects-2-sup/Supplementary figure 2.png]
